# A Protective Role for Complement C3 Protein during Pandemic 2009 H1N1 and H5N1 Influenza A Virus Infection

**DOI:** 10.1371/journal.pone.0017377

**Published:** 2011-03-09

**Authors:** Kevin B. O'Brien, Thomas E. Morrison, David Y. Dundore, Mark T. Heise, Stacey Schultz-Cherry

**Affiliations:** 1 Department of Medical Microbiology and Immunology, University of Wisconsin, Madison, Wisconsin, United States of America; 2 Department of Infectious Diseases, St. Jude Children's Research Hospital, Memphis, Tennessee, United States of America; 3 Department of Microbiology, University of Colorado–Denver, Aurora, Colorado, United States of America; 4 Department of Genetics, University of North Carolina, Chapel Hill, North Carolina, United States of America; 5 Department of Microbiology and Immunology, University of North Carolina, Chapel Hill, North Carolina, United States of America; 6 Carolina Vaccine Institute, University of North Carolina, Chapel Hill, North Carolina, United States of America; Institut Pasteur, France

## Abstract

Highly pathogenic H5N1 influenza infections are associated with enhanced inflammatory and cytokine responses, severe lung damage, and an overall dysregulation of innate immunity. C3, a member of the complement system of serum proteins, is a major component of the innate immune and inflammatory responses. However, the role of this protein in the pathogenesis of H5N1 infection is unknown. Here we demonstrate that H5N1 influenza virus infected mice had increased levels of C5a and C3 activation byproducts as compared to mice infected with either seasonal or pandemic 2009 H1N1 influenza viruses. We hypothesized that the increased complement was associated with the enhanced disease associated with the H5N1 infection. However, studies in knockout mice demonstrated that C3 was required for protection from influenza infection, proper viral clearance, and associated with changes in cellular infiltration. These studies suggest that although the levels of complement activation may differ depending on the influenza virus subtype, complement is an important host defense mechanism.

## Introduction

Since emerging in 1997, highly pathogenic avian influenza (HPAI) H5N1 viruses have been associated with over 500 human infections with an unprecedented fatality rate exceeding 60%. The significant virulence of these viruses, their continual evolution in birds, and the co-circulation of the pandemic H1N1 virus lead to concerns that the H5N1 viruses pose pandemic threats.

The severe disease associated with HPAI H5N1 infections in humans and animals could result from several factors including dissemination of the virus beyond the respiratory tract, higher and prolonged viral replication leading to increased viral cytolytic damage, differences in the host response induced by the H5N1 viruses, or a combination of all these factors. Although host responses are clearly complex, the clinical data and animal models suggest that the innate immune responses differ in H5N1 infected individuals (reviewed in [1], [2]). As compared to seasonal influenza infection H5N1 infected patients have elevated serum levels of several chemokines and cytokines [3], [4], [5]. Similar results were observed in animal models where H5N1 infection is associated with elevated cytokine and chemokine levels [2], [6], [7], [8], [9], enhanced recruitment of macrophages and neutrophils into the lungs leading to acute lung inflammation [10], and premature apoptosis of dendritic cells [11]. This aberrant host response is reminiscent of 1918 influenza virus infected animals [10], [12].

A major component of the innate immune response that has not been evaluated during HPAI H5N1 infections is complement. The complement system is comprised of more than 30 proteins responsible for recognizing and eliminating pathogens while stimulating early and late cellular functions (reviewed in [13]). Three biochemical pathways activate the complement system: the classical complement pathway, the alternative complement pathway, and the mannose-binding lectin pathway [14]. The hydrolysable C3 protein is the converging point for all three complement activation pathways, making it the central player in the complement cascade [15]. Upon activation, C3 is cleaved into C3a and C3 convertase, which supports the further cleavage of C5 into C5a. C3a and C5a function similar to chemokines promoting localized attraction and activation of immune cells including neutrophils, which serve an important role in early and late defense against pathogens including influenza virus [16], [17], [18], [19]. A recent study by Boon et al demonstrated a protective role for complement C5 in H5N1 influenza pathogenesis [20]. Thus, the goal of these studies was to fill our gap in knowledge by determining if mice infected with H5N1 influenza virus differed in complement C3 activation as compared to a seasonal or the pandemic 2009 H1N1 influenza virus and if C3 was required for protection from HPAI H5N1 influenza infection.

## Results

### H5N1 influenza virus increases C3 and C5a lung levels as compared to seasonal or pandemic influenza strains

**Figure 1:**
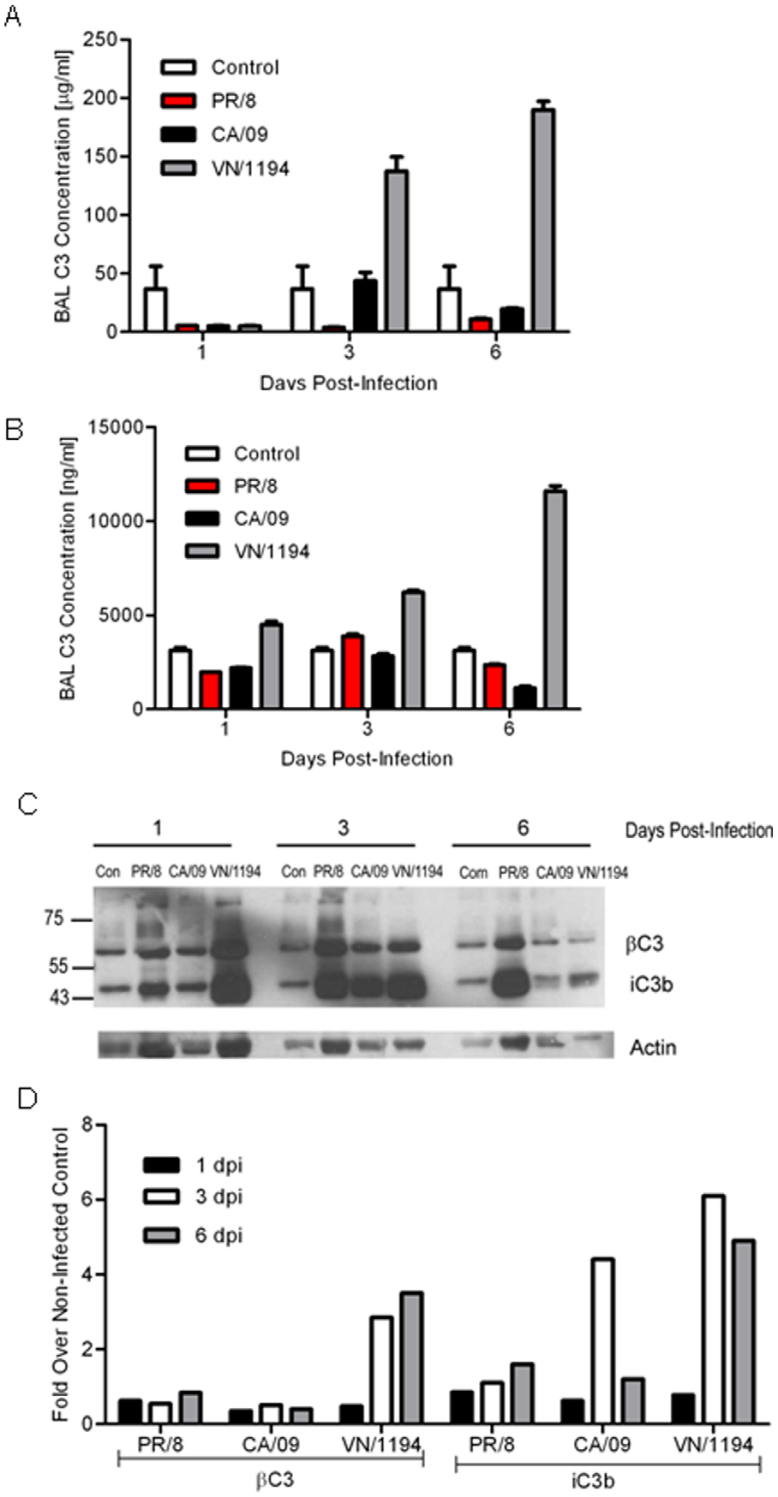
Complement levels in BAL during influenza infection. On days 1, 3, and 6 post-infection, BAL was collected from mice inoculated with PBS (control) or infected with PR/8, CA/09, or VN/1194 influenza virus and analyzed for complement C3 (A) or C5a levels (B) by sandwich ELISA. Error bars represent standard deviation. (C) C3 processing in the BAL of uninfected and influenza-infected mice at different times post-infection were analyzed by western blot analysis and results quantitated by densitometry (D). Results are representative of 2 separate experiments.

To quantitate C3 and C5a protein levels during influenza infection, C57BL/6 mice were lightly anesthetized and intranasally inoculated with PBS (uninfected control), seasonal H1N1 A/Puerto Rico/8/1934 (PR/8), pandemic 2009 H1N1 A/California/7/2009 (CA/09), or HPAI H5N1 A/Vietnam/1194/04 (VN/1194) and bronchioalveolar lavage (BAL) collected on days 1, 3, and 6 post-infection (pi). Using a mouse-specific C3 sandwich ELISA we found that within 3 days post-infection (dpi) C3 levels were >3-fold higher in the H5N1 VN/1194 virus infected mice as compared to controls and continued to increase to >4-fold by 6 dpi (Fig. 1A). This is in contrast to mice infected with seasonal (PR/8) or pandemic (CA/09) viruses where C3 levels never increased above control. A similar trend was observed for C5a. H5N1 VN/1194 infected mice had increased C5a levels within 3 dpi (∼2-fold) increasing to >4-fold by 6 dpi as compared to uninfected controls (Fig. 1B). There was no significant increase in PR/8 and CA/09 virus infected mice.

To monitor C3 processing in the BAL, immunoblotting was performed under reducing conditions with a polyclonal anti-C3 antibody and protein loading determined by anti-actin antibody ([21]; Fig. 1C and Fig. S1). To quantitate differences, densitometry was performed as compared to actin and results expressed as the fold change over uninfected controls (Fig. 1D). The anti-C3 antibody detected 2 major proteins at ∼65 and ∼43 kDa, which are likely βC3 and iC3b respectively. Mouse/rat C3 byproducts run at slightly different sizes than human [22], [23]. Similar to the C3 ELISA data, H5N1 VN/1194 infected animals had increased levels of both βC3 and iC3b in BAL at 3 and 6 dpi (Fig. 1C and D). Mice infected with seasonal PR/8 virus had levels at or below control uninfected throughout the course of infection. Comparable results were observed with the pandemic CA/09 infected mice except for a dramatic increase in iC3b levels at 3 dpi only. The reason for this is unclear and no increase was seen in the ELISA. Parallel trends were seen in BALB/c mice and mice infected with other strains of HPAI H5N1 virus suggesting that these results are not mouse or H5N1 VN/1194 strain specific (data not shown). In summation, these results suggest that H5N1 virus elicits higher levels of C5a and C3 in the lungs of infected mice.

### Increased morbidity and exacerbated inflammation in the lungs of C3^−/−^ infected mice

**Figure 2:**
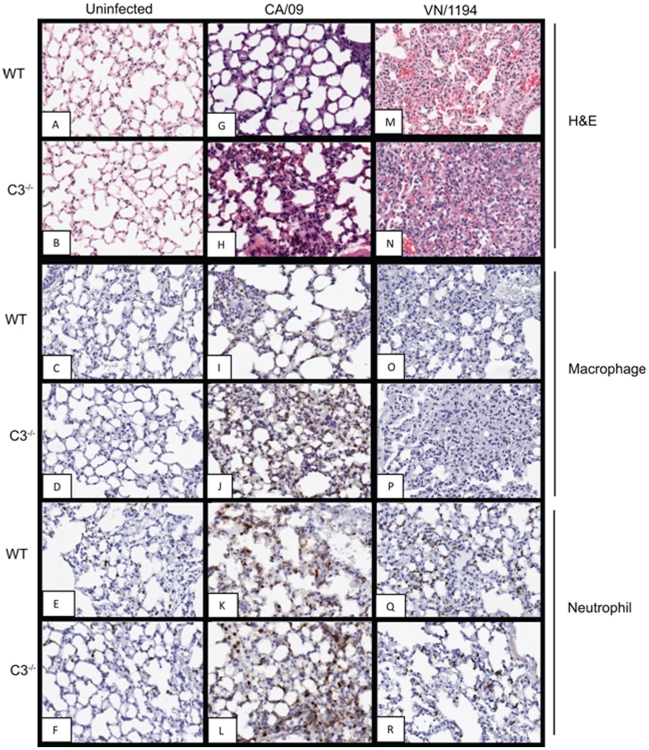
Increased inflammation and innate immune cells of influenza infected C3^−/−^ mice at 3 dpi. At 3 days post-infection, lung tissue was collected, formalin-fixed, and paraffin-embedded. Sections (4 µm thick) were stained with hematoxylin and eosin (A, B, G, H, M, N) or for macrophages (C, D, I, J, O, P), or neutrophils (E, F, K, L, Q, R). Representative pictures of each group are shown at 20× magnification.

**Figure 3:**
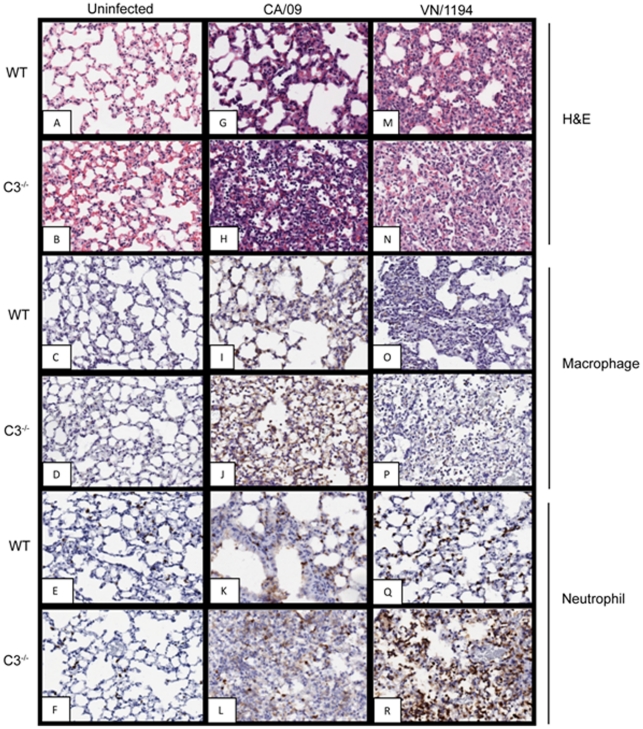
Increased inflammation and innate immune cells of influenza infected C3^−/−^ mice at 6 dpi. At 6 days post-infection, lung tissue was collected, formalin-fixed, and paraffin-embedded. Sections (4 µm thick) were stained with hematoxylin and eosin (A, B, G, H, M, N) or for macrophages (C, D, I, J, O, P), or neutrophils (E, F, K, L, Q, R). Representative pictures of each group are shown at 20× magnification.

**Figure 4:**
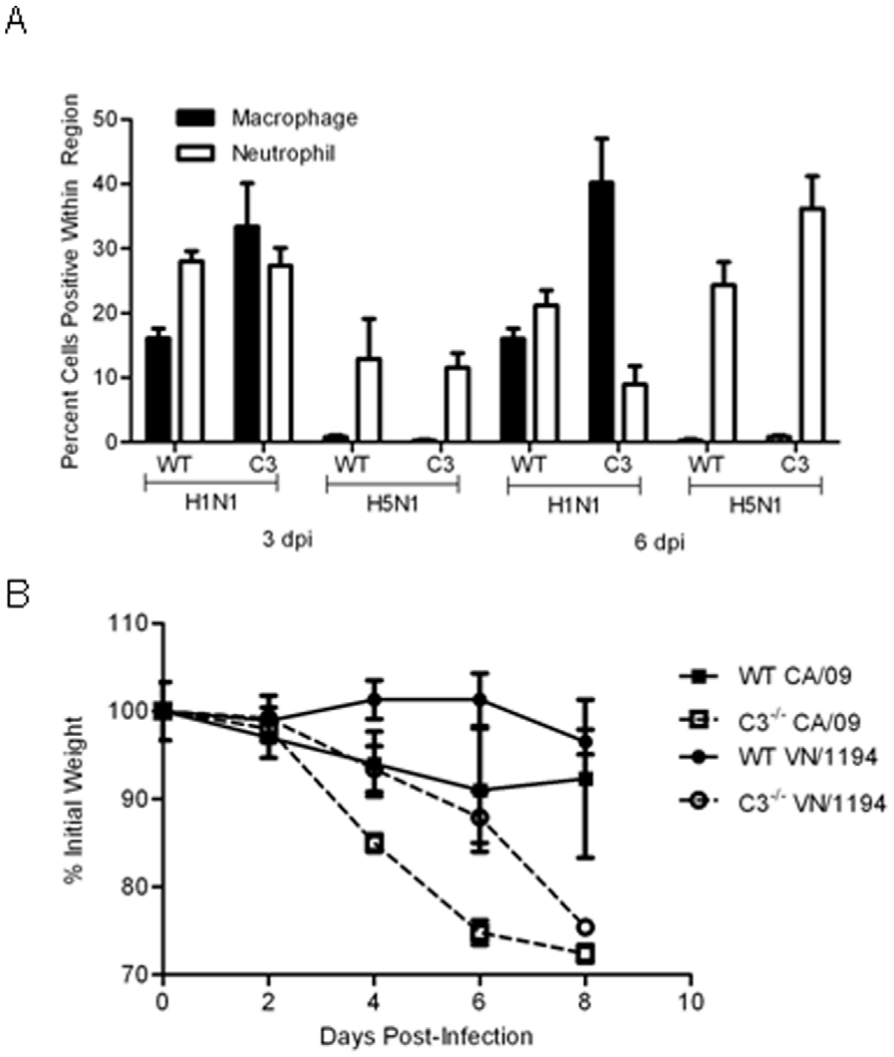
Increased morbidity in influenza infected C3^−/−^ mice. (A) Digital images of the slides was obtained and the percentage of positive nuclei in four random sections of the lung for each animal were determined with ImageScope using a nuclear-based algorithm. (B) C57BL/6 (WT) or C3^−/−^ mice were inoculated i.n. with equivalent MID_50_ units of CA/09 (10^5^ TCID_50_, n = 14) or VN/1194 (10^4^ TCID_50_, n = 14) influenza virus and weights monitored every 48 hpi (A). Error bars represent standard error deviation.

Increased complement has been associated with enhanced inflammation and tissue destruction [24]. Thus, we hypothesized that the elevated complement levels in the HPAI H5N1 infected mice could be involved in the prominent inflammatory response associated with these infections. To evaluate this, wild-type (WT) and C3 knockout mice (C3^−/−^) mice were intranasally inoculated with PBS (control), CA/09, or H5N1 VN/1194 virus, lungs collected at 3 (Fig. 2) and 6 dpi (Fig. 3) and histopathology performed. To evaluate the infiltrating cell population, tissues were stained for macrophages and neutrophils and quantitated by ImageScope. By 3 dpi, both the CA/09 (Fig. 2H) and VN/1194 (Fig. 2N) infected C3^−/−^ mice had enhanced inflammation as compared to WT (Fig. 2G and 2M) with the VN/1194 being more severe. In the CA/09 infected C3^−/−^ mice, this was associated with increased numbers of macrophages (Fig. 2J) as compared to WT (Fig. 2I). Quantitative analysis showed an increase from ∼17% positive cells in WT to ∼34% in C3^−/−^ mice (Fig. 4A). There was no change in neutrophil levels (Fig. 2K and L). Tissues from uninfected control animals are shown in Fig. 2A–F.

Although inflammation was more apparent in the H5N1 VN/1194 infected C3^−/−^ mice, this was not associated with significant differences in the numbers of macrophage or neutrophil as compared to WT (Fig. 2O–R). Both strains of mice had minimal numbers of macrophages and ∼10% staining for neutrophils (Fig. 4A). These numbers were significantly lower than those seen in CA/09 infected animals; regardless of the strain.

By 6 dpi, there was increased inflammation in the C3^−/−^ mice as compared to WT; including uninfected C3^−/−^ mice (Fig. 3B versus 3A). The CA/09 infected WT mice exhibited mild bronchiolitis and perivasculitis (Fig. 3G) comprised of both macrophages (Fig. 3I) and neutrophils (Fig. 3K). In contrast, the CA/09 infected C3^−/−^ mice had moderate to moderately severe bronchitis, bronchiolitis, and vasculitis, with perivasculitis, consolidation (Fig. 3H) and increased numbers of macrophages (Fig. 3J). Quantitative analysis showed an increase from ∼17% positive cells in WT to ∼42% in C3^−/−^ mice (Fig. 4A). What was most evident was the decrease in neutrophil numbers in the C3^−/−^ mice from ∼22% in WT to ∼10% in C3^−/−^ mice (Fig. 4A).

Although the pathologic changes were similar, the most prominent difference between VN/1194 infected WT and C3^−/−^ mice was the extent of the damage. VN/1194 infected WT mice had increased inflammation and damage in localized areas of the lung, primarily at the edges of the bronchi (Fig. 3M). In contrast, infected C3^−/−^ mice displayed a more diffuse, non-focal pathology with increased interstitial involvement (Fig. 3N). This was associated with an increase in neutrophils (Fig. 3R versus 3Q). Neutrophil levels increased from ∼25% in WT to ∼40% in C3^−/−^ mice (Fig. 4A). To determine if the changes in inflammation played a role in pathogenesis, infected mice were monitored for weight loss (Fig. 4B). By 8 dpi, the influenza infected C3^−/−^ mice had lost >25% of their day 0 weights (CA/09 *p*<0.009, VN/1194 *p*<0.05) and had to be humanely euthanized. In contrast, the infected WT mice never lost more than 8% of their day 0 weights (CA/09 *p*<0.005, VN/1194 *p*<0.0001).

### Delayed viral clearance in C3^−/−^ infected mice

**Figure 5:**
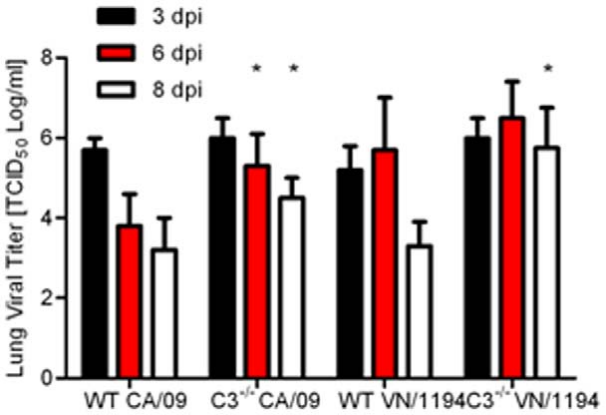
Delayed viral clearance in influenza infected C3^−/−^ mice. C57BL/6 (WT) or C3^−/−^ mice were inoculated i.n. with equivalent MID_50_ units of CA/09 (10^5^ TCID_50_, n = 14) or VN/1194 (10^4^ TCID_50_, n = 14) influenza virus. At 3, 6, and 8 days post-infection, lung homogenates were monitored for viral titers by TCID_50_ analysis on MDCK cells. Error bars represent standard deviation and asterisk (*) indicates significant increase in viral titers as compared with WT infected mice.

Finally to better understand the mechanism of C3-mediated protection, viral titers were measured in the lungs on days 3, 6, and 8 pi (Fig. 5). In CA/09-infected WT mice, lung titers were highest 3 dpi (10^6.5^ TCID_50_/ml) and decreased to approximately 10^3.5^ TCID_50_/ml by 8 dpi (Fig. 5). In contrast, the C3^−/−^ infected mice had significantly higher viral titers at days 6 (*p*<0.04) and 8 pi (*p*<0.03) suggesting a delay in viral clearance. Similar trends were observed in the VN/1194-infected C3^−/−^ mice, although these differences were not significant until 8 dpi when the viral titers in WT mice were 10^3.5^ TCID_50_/ml as compared to 10^5.75^ TCID_50_/ml (*p*<0.02) in C3^−/−^ infected mice. Overall, these studies suggest that C3 is an important host response during influenza infection; potentially by aiding in viral clearance and regulating lung inflammation.

## Discussion

Here, we present data that the complement component C3 is required for protection from pandemic 2009 H1N1 and HPAI H5N1 influenza virus infections by aiding in viral clearance and regulating lung inflammation. Our work complements previous studies demonstrating a protective role for C3 against mouse-adapted strains of influenza virus [25], [26], [27]. These studies demonstrated that complement C3 was important for T-cell priming and migration to the lung [27] and promoting the expansion of CD8+ and CD4+ T cells during infection [28]. The lack of T cell priming, expansion and migration in the C3^−/−^ mice could explain the delayed viral clearance.

The complement system is a major component of innate immunity and consists of both soluble factors and cell surface receptors that interact to sense and respond to invading pathogens. Three general activation pathways, referred to as the classical, alternative, and lectin pathways, converge on C3 (composed of an alpha and beta chain), the central component of the complement system. Formation of C3 convertases leads to cleavage of C3 to its active fragments, the anaphylatoxin C3a and the opsonin C3b. This cleavage event exposes a reactive thioester that allows covalent attachment of C3b to target surfaces. C3b can be further cleaved into the signaling fragments iC3b, C3dg, and C3d which regulate phagocytosis and a variety of other immune cell effector functions. In addition, C3b binds the C3 convertases resulting in a substrate change from C3 to C5, which is cleaved to the anaphylatoxin C5a and the initiator of the membrane attack complex C5b.

Although it was clear that complement component C3 was required for host defense, the levels of C3 and complement activation fragments in BAL differed amongst the different influenza virus infections. We failed to detect any significant increase in total C3 levels in the BAL of PR/8 or 2009 pandemic CA/09 virus infected mice as compared to uninfected controls using a mouse-specific C3 sandwich ELISA. In contrast, increased levels of total C3 were detected in the BAL of mice inoculated with H5N1 VN/1194. Increased levels of C5a, a marker of complement activation and a potent anaphylatoxin, were also detected in the BAL of H5N1 VN/1194 inoculated mice, but not mice inoculated with either PR/8 or CA/09. To further investigate activation of the complement system by the different influenza virus infections, immunoblot analyses of BAL were performed. Consistent with the C3 and C5a ELISA data, increased levels of the C3 beta chain, which is not proteolytically processed and serves as a marker for total C3 levels, and iC3b, which is derived from the proteolysis of the C3 alpha chain and serves as a marker of complement activation, were detected in BAL collected from mice inoculated with H5N1 VN/1194. In addition, immunoblot analyses detected an increase in iC3b levels at 3 dpi in BAL collected from CA/09 virus infected mice. Taken together, these results suggest that CA/09 and H5N1 VN/1194 are more powerful activators of the complement system compared to PR/8 infection. The reasons for the differences amongst the viruses and the viral genes involved are under investigation.

Increased complement activation can be associated with enhanced viral pathogenesis. For example, compared to WT animals, complement-depleted mice infected with Sindbis virus had less morbidity and mortality despite increased viral replication and spread, suggesting a potentially pathologic role for complement [29], [30]. Similar results occurred with the arthritogenic alphavirus Ross River Fever virus in which complement-deficient mice exhibited far less severe disease signs and tissue damage than WT mice despite similar viral titers [24], [31], [32]. Because of this, we hypothesized that the increased complement levels in the HPAI H5N1 infected mice could be associated with the enhanced inflammation associated with these infections. Pathological examination demonstrated that the H5N1 VN/1194 infected WT mice had enhanced inflammation as compared to the pandemic 2009 H1N1 CA/09 infection within 3 dpi. However, the inflammation was exacerbated in the infected C3^−/−^ animals suggesting that C3 plays a protective role during influenza infection.

In summary, the results of our studies suggest that complement contributes to protection against 2009 pandemic H1N1 and HPAI H5N1 influenza infection in mice. Given that complement polymorphisms are common and can be associated with increased susceptibility to several infectious diseases [33], [34], [35], [36], it would be interesting to determine the state of complement activation during influenza infection in humans and assess the role in disease severity.

## Materials and Methods

### Ethics statement

All procedures involving animals were approved by the University of Wisconsin-Madison School of Medicine and Public Health, and the St. Jude Children's Research Hospital IACUC's and was in compliance with the Guide for the Care and Use of Laboratory Animals. These guidelines were established by the Institute of Laboratory Animal Resources and approved by the Governing Board of the U.S. National Research Council.

### Laboratory facilities

All experiments using H5N1 viruses were conducted in a Biosafety level 3 enhanced containment laboratory [37]. Investigators were required to wear appropriate respirator equipment (RACAL, Health and Safety Inc., Frederick, MD). Mice were housed in HEPA-filtered, negative pressure, vented isolation containers.

### Viruses and cells

A/Puerto Rico/8/34 (PR/8) and A/California/04/2009 (CA/09, provided by Dr. Jacco Boon, St. Jude Children's Research Hospital) H1N1 viruses were propagated in the allantoic cavity of 10-day-old specific pathogen-free embryonated chicken eggs at 37°C. Allantoic fluid was harvested, clarified by centrifugation and stored at −70°C. A/Vietnam/1194/2004 (VN/1194) (provided by Dr. Alexander Klimov; CDC, Atlanta, Georgia) was propagated in Madin Darby canine kidney (MDCK) cells as described [38]. MDCK cells were cultured in Eagle's minimum essential medium supplemented with 4.5 g glucose per liter, 2 mM glutamine (Mediatech), and 10% FBS (Gemini BioProducts, West Sacramento, CA) and grown at 37°C under 5% CO_2_.

### Viral titers

Viral titers were determined by tissue culture infectious dose 50 (TCID_50_) assays on MDCK cells as described [38] and quantitated by Reed and Muench analysis [39]. Results are expressed as the mean log_10_ TCID_50_/ml.

### Animal experiments

10–12 week of age female C57BL/6J (WT) or C3^−/−^ (B6.129S4-*C3^tm1Crr^*/J) mice were purchased from The Jackson Laboratory (Bar Harbor, ME). For infection, mice were lightly anesthetized using isofluorane and intranasally (in) inoculated with PBS or equivalent mouse infectious dose 50 (MID_50_) of PR/8 (1×10^4^ TCID_50_), CA/09 (1×10^5^ TCID_50_), or VN/1194 (1×10^4^ TCID_50_) in 25 µl PBS. Mice were monitored daily for clinical signs of infection [40] and weighed every 48 hpi. At different times post-infection, two control and three infected mice were anesthetized, terminally bled, and lungs harvested. The large lung lobe was washed and immediately stored in 10% buffered formalin for histological analysis. The remaining lobe was homogenized in 1 ml PBS and viral titers determined by TCID_50_ analysis.

### Histopathology and immunohistochemical staining

Tissues were fixed in 10% neutral buffered formalin solution, processed, and paraffin embedded. Four micron thick sections were stained with hematoxylin and eosin or for macrophages (Mac2/Galectin 2, 1∶1000, Abcam, Cambridge, MA) or neutrophils (NeuN, 1∶1000, Abcam) by the St. Jude Veterinary Pathology Core Facility. Digital images were obtained with ScanScope (Aperio, Vista, CA) and the percentage of positive nuclei in four random sections of the lung for each animal were determined with ImageScope using a nuclear-based algorithm.

### Complement component C3 and C5a ELISA

Protein levels were determined in BAL by BCA Protein Assay (Pierce, Rockford, IL) and C3 and C5a levels were quantitated by sandwich ELISAs (GenWay Biotech Inc, San Diego, CA and R&D Systems, Minneapolis, MN respectively) using equivalent protein concentrations following manufacturer's instructions.

### Western blot

Equivalent volumes (30 µl) of BAL were separated by SDS-PAGE under reducing conditions and transferred to a nitrocellulose membrane using the iBlot Dry Blotting system (Invitrogen, Carlsbad, CA). The membrane was blocked with 5% dry milk in TBS containing 0.1% Tween-20 (TTBS), and probed with goat anti-mouse C3 antibody (1∶1000; Cappel) or goat anti-actin antibody (1∶1000 Santa Cruz) overnight at 4°C. After extensive washes, membranes were incubated with a donkey anti-goat HRP-conjugated antibody (1∶10,000; Southern Biotechnology, Birmingham, AL) for 1 h at room temperature, and signal was detected using the SuperSignal West Pico Chemiluminescent Substrate (Pierce). Densitometry was performed on scanned immunoblot images using the ImageJ gel analysis tool. The gel analysis tool was used to obtain the absolute intensity (AI) for each experimental complement band and corresponding actin control band. The ratios for each experimental band was calculated by dividing the corresponding control AI by the experimental AI to determine the fold change as compared to the uninfected control samples.

### Statistical analyses

Statistical significance of data was determined by using analysis of variance (ANOVA) or Student's *t*- test on GraphPad Prism (San Diego, CA). All assays were run in triplicate and are representative of at least 2 separate experiments. Error bars represent standard deviation and statistical significance was defined as a *p* value of less than 0.05.

## Supporting Information

Figure S1 **Complement activation products in BAL during influenza infection.** On days 1, 3, and 6 post-infection, BAL was collected from mice inoculated with PBS (control) or infected with PR/8, CA/09, or VN/1194 influenza virus and analyzed for complement C3 activation products by western blot analysis. Results are representative of 2 separate experiments.
(TIF)
