# Supplementary figures and images for: A Protective Role for Complement C3 Protein during Pandemic 2009 H1N1 and H5N1 Influenza A Virus Infection
Source: PLoS One. 2011 Mar 9;6(3):e17377. doi: 10.1371/journal.pone.0017377 (PMC3052313; doi:10.1371/journal.pone.0017377)

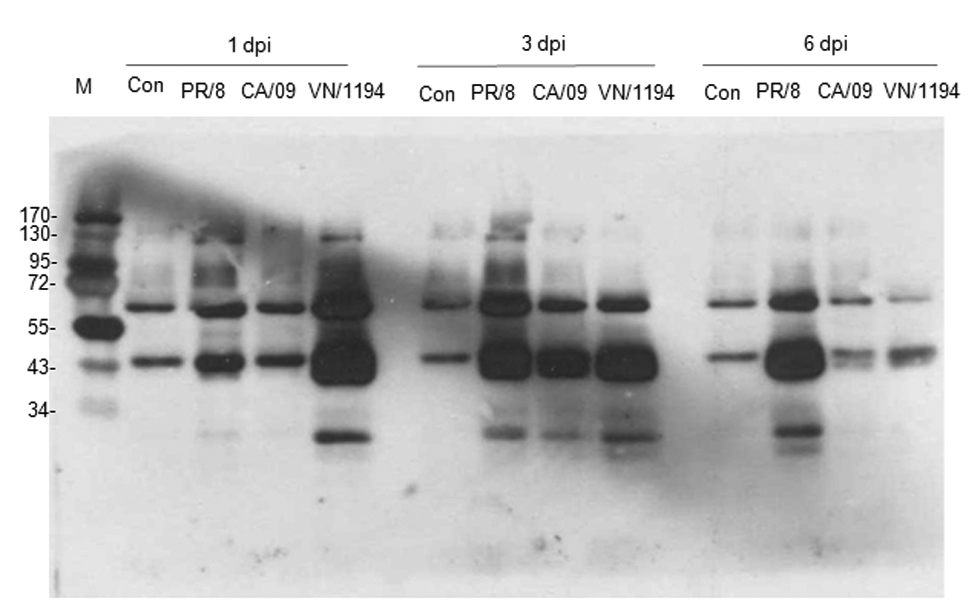

Supplement: Figure S1 — Complement activation products in BAL during influenza infection. On days 1, 3, and 6 post-infection, BAL was collected from mice inoculated with PBS (control) or infected with PR/8, CA/09, or VN/1194 influenza virus and analyzed for complement C3 activation products by western blot analysis. Results are representative of 2 separate experiments. (TIF) [file pone.0017377.s001.tif]
